# Association of the Waist-to-Height Ratio with Cardiovascular Risk Factors in Children and Adolescents: The Three Cities Heart Study

**Published:** 2010

**Authors:** Robespierre C. Ribeiro, Mário Coutinho, Marco A Bramorski, Isabela C. Giuliano, Júlia Pavan

**Affiliations:** 1 Department of Pediatrics, School of Medicine, Federal University of Minas Gerais, Minas Gerais, Brazil; 2 Department of Cardiology, School of Medicine, Federal University of Santa Catarina, Florianópolis, Brazil; 3 Department of Statistics, University of São Paulo, São Paulo, Brazil

**Keywords:** Anthropometry, Cardiovascular risk factors, Multicenter study, Obesity, Pediatrics, Brazil

## Abstract

**Objectives:**

To determine the best anthropometric index in relation to cardiovascular disease risk factors among children and adolescents.

**Methods:**

This cross-sectional school-based study was conducted among a random sample of 3179 students, aged 6 to 18 years, in three large cities in Brazil.

**Results:**

The prevalence of overweight and obesity was 10% and 5%, respectively. In relation to the students in the lower quartile (Q1) of the distribution of subscapular skinfold, the students in the upper quartile (Q4) presented a 2.0 times higher risk (odds ratio) of having elevated total cholesterol levels. Overweight and obese students had a 3.3 times higher risk of having elevated systolic blood pressure, and a 1.9 times higher risk of elevated diastolic blood pressure than other students. The less active students presented a 1.58 times higher risk of having waist-to-height ratio (WHtR) above the upper tertile (Q3). WHtR mean values was 0.46 (SE 0.00) presented the largest area under the curve (AUC) [0.613 (CI995%:0.578-0.647)] for high total cholesterol levels, [0.546 (CI995%: 0.515-0.578)] for low HDL-C levels, and [0.614 (CI95%: 0.577-0.651)] for high LDL-C levels, while body mass index presented the largest AUC [0.669 (CI95%: 0.64-0.699)] for increased diastolic blood pressure followed by the waist circumference for increased systolic blood pressure [0.761 (CI95%: 0.735-0.787)].

**Conclusions:**

WHtR is considered as a simple and accurate anthropometric parameter that identifies youth with cardiovascular risk factors. In this study, WHtR above 0.44 was indicative of risk factors in children and adolescents. These findings can be applied in future preventive strategies against CVDs, and screening programs.

## INTRODUCTION

Cardiovascular diseases (CVD) are the leading cause of death and loss of disability-adjusted life years in the world, with about of 80% of its burden occurring in low-income and middleincome countries.^1^,^2^ Although the occurrence of this huge burden in these nations, most cardiovascular research is being conducted in developed countries with largely white, European/American populations, and specific lifestyle and socio-cultural backgrounds, making it difficult to assume whether the findings of these researches apply to populations in developing nations or not. There are data suggesting that risk factors for coronary heart disease vary between ethnic populations.^3^,^4^ In Brazil, as else where in the world, the increasing prevalence of obesity among adults^5^,^6^ raises concerns about trends in CVD mortality rates. The long-term decline in these rates has leveled off over the last ten years however; the increasing rate of obesity prevalence will probably increase these mortality rates.^7^ An important component of adult obesity is excess weight gain during childhood and adolescence, which increases the burden of cardiovascular risk later in life.^8^ Pediatricians and health care managers who have traditionally focused on undernutrition among children must now also be on the alert for overnutrition among their patients.^9^ Identifying children who are at risk of overweight and obesity, however, poses some diagnostic problems. The determination of skin-fold indices, waist circumference, and the correlation between weight and height, i.e., body-mass index (BMI), all have important limitations.^10^–^15^

Various anthropometric indices of obesity have been suggested to predict CVDs. According the type of obesity they are intending to measure BMI reflecting overall obesity, skin-fold thickness for assessing regional obesity, while waist circumference (WC), waist-to-hip ratio (WHR), conicity index – waist-to-height ratio, all these last three measures indicate abdominal fat deposition.^11^–^15^

Although BMI is the most studied index, being significantly related to CVD risk factors as demonstrated by cross-sectional and prospective studies,^1^–^8^ there are increasing doubt about its role in predicting CVD risk factors, which has lead to an increasing evidence for abdominal obesity indices such as WC, WHtR, and waist-to-height ratio (WHtR) as predictors of CVD among other new indices that are being suggested from time to time.^14^ A computed tomography study demonstrated that WHtR showed the highest correlation with intra-abdominal fat, compared to BMI, WC and WHR. 16

As there are scarce studies about anthropometric measures in Latin-American children and adolescents, the current study was designed to determine the best anthropometric index in relation to metabolic CVD risk factors among children and adolescents in Brazil, as a society undergoing a profound shift from undernutrition to overnutrition.

## METHODS

A cross-sectional study of CVD risk factors was performed in 1998-99 in the city of Belo Horizonte, the third largest city in Brazil, and in two other large cities, Florianópolis^17^ and Blumenal in 2001. The design and methods were similar in the three studies since they were coordinated by the Belo Horizonte Heart Study^18^ main investigator. A two-stage cluster sampling plan was used to select the samples. First, the schools were randomly selected among the public and private city schools. Within each school, classrooms were randomly selected, then all students from those classrooms included in the sample. At the end of this process the overall sample had 3179 students, between the ages of 6 to 18 years. For this kind of plan one had to select the sample size for the first and second stage units. In our case, we followed the recommendations of Kish,^19^ and fixed the α and β error as 0.05 and 0.20, respectively. We also used the prevalence of high blood pressure levels as the main outcome for the specification of the sample plan.^20^ All students were invited to complete a questionnaire, and to undergo measurements of serum lipids, blood pressure and physical examination. All tests and measurements were performed on site at the respective schools. Prior to data collection, the questionnaire was tested in two schools in each city, to ensure that its contents and response format were appropriate for the students/parents. Institutional review board approval was obtained from all institutions involved in the study for collection of data.

Because the number of Asian and native students was too small, only reports from white, mixed race, and black students were included. For analyses purposes, we collapsed whites and mixed-race students into a single category, as they showed no significant statistic difference in the modeling process. Child (<10 years of age) and adolescent (10-19 year-old) were defined according to the World Health Organization.^21^ Puberty criteria was not used for definitions of these age strata as it would cause moral constrains at physical examinations, mainly in some randomized religious school girls, and also because of logistic concerns. Only children from school age (≥ 6 years of age) were included in the study.

Anthropometric measurements included weight, height, waist and hip circumferences, triceps, subscapular, and suprailiac skinfold thickness. All measurements followed standard procedures.^22^ Participants were examined dressed in light clothing and barefoot. Height was measured to the nearest 0.1 cm using a portable stadiometer and weight to the nearest 0.1 kg. Skinfold thickness was recorded to the nearest 1mm by Lange skinfold caliper (Cambridge Scientific, Cambridge, MA). The means of these multiple anthropometric measurements were used for all analyses. BMI was calculated as weight in kilograms divided by height in meters squared. Age was computed from the reported birth date. To assess the prevalence of childhood obesity in this population and to infer risk of subsequent obesity-related disease, a modest excess weight, BMI-for-age and gender percentile ≥ 85%, using the IOTF definition of overweight, was used as the cutpoint defining excess weight, since it is associated with higher levels of metabolic markers of insulin resistance in boys and girls.^23^,^24^ To assess the adiposity body distribution (truncal adiposity), the upper quartile (>Q3) of skinfolds, WC, WHR and WHtR, sample distributions, were compared to the lower quartile of these variables distributions.

The lipid variables examined were total, LDL and HDL cholesterol. They were recorded in milligrams per deciliter, which can be converted to millimoles by multiplying by 0.02586. According to the National Heart, Lung, and Blood Institute, the following cut-points for desired HDL- cholesterol levels were established for children and adolescents: above 40 mg/dL for those below 10 years of age and above 35 mg/dL for those with 10-19 years of age. For elevated total cholesterol and LDL-C levels, cut-points were established above 200 mg/dL and 130 mg/dL respectively. 25

Blood pressure was considered “high normal” (borderline) when the systolic or diastolic pressure was between the 90^th^ and 95^th^ percentiles for the reference population; significant high blood pressure was defined as a systolic or diastolic pressure above the 95^th^ percentile, and severe high blood pressure as a systolic or diastolic pressure above the 99^th^ percentile for the reference population, or approximately 10 mmHg above the 95^th^ percentile in accordance with the National High Blood Pressure Education Program Working Group on Hypertension Control in Children and Adolescents.^26^ We considered “blood pressure higher than normal” or “high blood pressure” when the systolic or diastolic levels were above the 95^th^ percentile for the reference population.

Race was considered only by identification of skin color. Socioeconomic status was based on the Brazilian Association of Market Research Institutes scores based on a validated questionnaire that asked about ownership of a home, car, household appliances such as a refrigerator, washing machine, etc.^27^ This five-category score questionnaire was collapsed into two categories of high and low socioeconomic strata.

### 

#### Statistical analysis:

The analyses were performed with the SPSS for Windows (Release 8.0, Chicago, IL, USA) and Statistical Analysis Software – SAS (Release 8.02, SAS Institute Inc, Cary, NC, USA), on a PC plataform.

All tests were based on the 0.05 level of significance. Univariate analyses for testing between group variable differences were done using the independent sample t test and chisquare test of independence for continuous and discrete variables, respectively.

For the best anthropometric index determination, partial correlations were first performed between CVD risk factors and anthropometric indices, without adjusting for age and gender since WHR does not vary with this demographic variables.^28^ A Receiver Operator Characteristic (ROC) curve analyses were used to calculate the area under ROC curves (AUC) between each CVD risk factor, i.e. clinical variables, and anthropometric index. As a plot of the sensitivity against 1-specificity for each cutoff value, AUC is an indicator of how good the anthropometric indices can distinguish a positive test outcome. Each value of an anthropometrical parameter was used as a cutoff value to calculate its sensitivity and specificity in classifying a CVD risk factor, and optimal cutoff value was denoted by the value, point on the curve, which had the largest sum of sensitivity and specificity together with less false-positives results (1-Specificity).^29^,^30^ Moreover, by applying logistic regression models, adjusted odds ratios (OR) was calculated as an estimate of the chances of adverse CVD risk factor condition, with each CVD risk factor as dependant variables. Statistical inference was based on 95%. All regression analyses were controlled for various confounding factors including skin color and age.

## RESULTS

Tables 1, 2 and 3 show the mean values and standard error (SE) of the studied variables according to gender, age group and race.

**Table 1:** Characteristics of participants according to gender: the Three Cities Heart Study

Variables		Mean (SE)	Total	n		
	
	Female	Male		Female	Male	Total
Total cholesterol (mg/dL)	163.80 (0.69)	156.60 (0.74)	160.61 (0.51)	1724	1375	3106
LDL-C(mg/dL)	95.87 (0.61)	90.17 (0.64)	93.35 (0.45)	1724	1373	3104
HDL-C(mg/dL)	50.10 (0.26)	47.81 (0.29)	49.07 (0.19)	1724	1374	3105
Systolic blood pressure (mmHg)	110.19 (0.33)	112.03 (0.43)	110.52 (0.27)	1726	1386	3119
Diastolic blood pressure (mmHg)	67.56 (0.26)	67.42 (0.33)	67.54 (0.21)	1726	1386	3119
Subscapular skinfold(mm)	12.59 (0.15)	10.10 (0.17)	11.49 (0.12)	1733	1389	3129
Suprailiac skinfold(mm)	15.04 (0.19)	11.72 (0.24)	13.56 (0.15)	1733	1389	3129
Tricipital skinfold(mm)	15.54 (0.14)	12.02 (0.16)	13.97 (0.11)	1733	1389	3129
Sum of skinfolds(mm)	4316 (0.45)	33.84 (0.54)	39.03 (0.36)	1733	1389	3129
Waist circumference(cm)	67.96 (0.24)	69.16 (0.30)	68.52 (0.19)	1732	1391	3130
Waist-to-hip ratio	0.81 (0.00)	0.85 (0.00)	0.83 (0.00)	1731	1391	3129
Body mass index	19.32 (0.08)	19.13 (0.10)	19.24 (0.06)	1734	1395	3136
Waist-to-height ratio	0.45 (0.00)	0.45 (0.00)	0.45 (0.00)	1731	1391	3129

**Table 2:** Characteristics of participants according to age group: the Three Cities Heart Study

Variables		Mean (SE)	Total	n		
	
	Child	Adolescent		Child	Adolescent	Total
Total cholesterol (mg/dL)	165.06 (0.80)	157.79 (0.66)	160.61 (0.51)	1200	1899	3106
LDL-C(mg/dL) 97.58 (0.70)	90.67 (0.57)	93.35 (0.45)	1199	1898	3104
HDL-C(mg/dL)	48.36 (0.30)	49.54 (0.25)	49.07 (0.19)	1199	1899	3105
Systolic blood pressure (mmHg)	103.85 (0.39)	114.62 (0.33)	110.52 (0.27)	1205	1907	3119
Diastolic blood pressure (mmHg)	64.12 (0.32)	69.63 (0.26)	67.54 (0.21)	1205	1907	3119
Subscapular skinfold(mm)	9.76 (0.18)	12.57 (0.15)	11.49 (0.12)	1210	1912	3129
Suprailiac skinfold(mm)	11.41 (0.24)	14.92 (0.20)	13.56 (0.15)	1210	1912	3129
Tricipital skinfold(mm)	13.00 (0.16)	14.59 (0.15)	13.97 (0.11)	1210	1912	3129
Sum of skinfolds(mm)	34.17 (0.54)	42.08 (0.45)	39.03 (0.36)	1210	1912	3129
Waist circumference(cm)	62.07 (0.26)	72.56 (0.21)	68.52 (0.19)	1211	1912	3130
Waist-to-hip ratio	0.85 (0.00)	0.82 (0.00)	0.83 (0.00)	1211	1911	3129
Body mass index (Kg/m2)	17.38 (0.09)	20.41 (0.08)	19.24 (0.06)	1214	1915	3136
Waist-to-height ratio	0.46 (0.00)	0.45 (0.00)	0.45 (0.00)	1210	1912	3129

**Table 3:** Characteristics of participants according to skin color: the Three Cities Heart Study

Variables		Mean (SE)	Total	n		
	
	White	Black		White	Black	Total
Total cholesterol (mg/dL)	161.11 (0.53)	156.20 (1.87)	160.61 (0.51)	2735	307	3106
LDL-C(mg/dL)	93.43 (0.46)	92.31 (1.65)	93.35 (0.45)	2734	306	3104
HDL-C(mg/dL)	49.23 (0.20)	47.93 (0.64)	49.07 (0.19)	2734	307	3105
Systolic blood pressure (mmHg)	110.31 (0.29)	112.31 (0.83)	110.52 (0.27)	2747	308	3119
Diastolic blood pressure (mmHg)	67.34 (0.22)	69.16 (0.64)	67.54 (0.21)	2747	308	3119
Subscapular skinfold(mm)	11.44 (0.12)	11.80 (0.34)	11.49 (0.12)	2757	308	3129
Suprailiac skinfold(mm)	13.51 (0.16)	13.82 (0.49)	13.56 (0.15)	2216	849	3129
Tricipital skinfold(mm)	14.00 (0.12)	13.62 (0.35)	13.97 (0.11)	2757	308	3129
Sum of skinfolds(mm)	38.94 (0.38)	39.24 (1.10)	39.03 (0.36)	2757	308	3129
Waist circumference(cm)	68.45 (0.20)	68.95 (0.64)	68.52 (0.19)	2759	307	3130
Waist-to-hip ratio	0.83 (0.00)	0.82 (0.00)	0.83 (0.00)	2759	307	3129
Body mass index (Kg/m2)	19.19 (0.07)	19.70 (0.22)	19.24 (0.06)	2759	307	3129
Waist-to-height ratio	0.45 (0.00)	0.44 (0.00)	0.45 (0.00)	2758	307	3129

The results revealed that mean values of total cholesterol for female students were 7.2 mg/dL higher than those of the male students. The same tendency was observed for the LDL-C and HDL-C fractions, with LDL-C levels approximately 5.7 mg/dL higher for females than for males and HDL-C approximately 2.3 mg/dL higher for females than for males. Except for HDL-C, children presented higher mean values of serum lipids than adolescents, with total cholesterol levels approximately 7.3 mg/dL higher for children than for adolescents, LDL-C levels approximately 7.0 mg/dL higher for children than for adolescents, and HDL-C approximately 1.2 mg/dL higher for adolescents than for children. Lower mean values of serum lipids were detected in the black students in comparison to their white counterparts. These differences were 4.9 mg/dL for total cholesterol, 1.2 mg/dL for HDL-C and 1.3 mg/dL for LDL-C levels.

Blood pressure mean values were higher in male adolescent black students compared to female children white students. The skinfold mean values were higher in females, higher in adolescents and without difference between white and black students. The other measures of adiposity distribution, WHR and WHtR, showed no significant differences in terms of gender, and were slightly higher values in children and black students. BMI mean values were similar in both genders and races, but were higher in adolescents than in children.

In relation to the ranges classified as “desir-able”, “borderline” and “elevated” lipid values,^25^ more than one third (36.0%) of participants presented total cholesterol levels higher than the values considered desirable (> 170 mg/dL), and almost one quarter (23.0%) also presented LDL-C levels higher than the values considered desirable (> 110 mg/dL). Considering HDL-C, approximately 11% of the students presented values considered as undesirable (< 10 years: < 40 mg/dL; 10-19 years: < 35 mg/dL).

The frequency of overweight as determined by BMI-for-age between the 85^th^ to 94^th^ percentiles was 10%, while the frequency of obesity as determined by BMI-for-age equal or greater than the 95th percentile was 5%, and 15% for excess weight (BMI > 85th percentile).

We found that 12% of the students presenting higher than normal blood pressure readings (systolic and/or diastolic > 90^th^ percentile).

Table 4 shows weak correlation values between each of the nine anthropometric indices with clinical variables, and moderate to strong and significant (p < 0.01) correlation values between the anthropometric indices.

**Table 4:** Estimated partial correlation coefficients values between variables: the Three Cities Heart Study

	Total Cholesterol	HDLColesterol	LDLCholesterol	Diastolic blood pressure	Systolic blood pressure	Subscapular skinfold	Suprailiac skinfold	Triceps skinfold	Skinfolds sum	Waist circumference	Hip circumference	Waist to hip circumference	Body mass index	Waist-to-height ratio
Total Cholesterol	-													
HDL-Colesterol	0.37	-												
LDL-Cholesterol	0.90	0.09	-											
Diastolic blood pressure	-0.01	-0.08	0.03	-										
Systolic blood pressure	-0.05	-0.07	-0.03	0.67	-									
Subscapular skinfold	0.15	-0.08	0.16	0.24	0.29	-								
Suprailiac skinfold	0.13	-0.07	0.14	0.26	0.30	0.82	-							
Tricep skinfold	0.15	-0.02	0.16	0.21	0.21	0.80	0.80	-						
Skinfolds sum	0.15	-0.06	0.17	0.26	0.29	0.93	0.95	0.92	-					
Waist circumference	0.04	0.00	0.01	0.31	0.45	0.64	0.57	0.49	0.61	-				
Waist-to-hip circumference	0.04	-0.03	0.02	0.03	0.05	0.05	-0.01	-0.03	0.01	0.37	-0.32	-		
Body mass index	0.07	-0.04	0.06	0.29	0.42	0.76	0.70	0.64	0.75	0.82	0.82	0.03	-	
Waist-to-height ratio	0.17	-0.04	0.15	0.11	0.15	0.60	0.51	0.50	0.57	0.71	0.28	0.57	0.61	-

**Figure 1:**
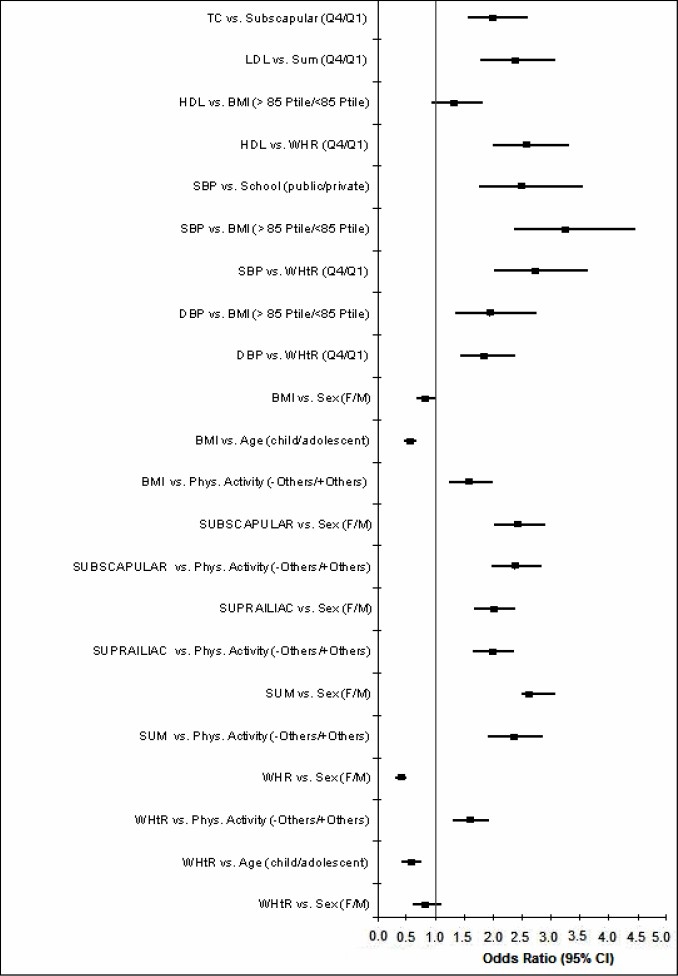
Odds ratio (logistic regression) for cardiovascular risk factors in children and adolescents.

Table 5 and Figure 1 show the odds ratio (OR) for CVD risk factors in children and adolescents. The OR of a student with increased subscapular skinfold thickness to have high cholesterol levels (>200mg/dL) was 2.01 (CI95%: 1.57-2.59) higher than those without it. Students considered being “less active than the others” presented 2.36 (1.98-2.82) times higher odds to have increased values of this skinfold than those who are more active. Moreover, participants who were “less active than the others” presented 1.97 (1.65-2.35) and 2.34 (1.92-2.85) times respectively more odds to have increased suprailiac skin fold thickness and sum of the three skinfold thickness than those who were more active.

**Table 5:** Results of logistic regression analysis of variables studied and cardiovascular risk factors in children and adolescents: the Three Cities Heart Study

Variable	Ratio	Odds	95% Confidence Interval	
			Lower	Higher
	**Elevated total cholesterol§**			
Subscapular skinfold thickness	Q4 / Q1†	2.01	1.57	2.59
	**Elevated LDL-C§**			
Sum of skinfolds^3^	Q4 / Q1	2.34	1.79	3.06
	**Undesirable HDL-C§**			
Body mass index	>85^th^ vs <85^th^ percentile	1.95	1.46	2.62
Waist-to-hip ratio	Q4 / Q1	2.56	1.99	3.29
	**Elevated systolic blood pressure¶**			
Type of school	public vs. private	2.48	1.75	3.54
Body mass index	>85^th^ vs. <85^th^ percentile	3.25	2.38	4.45
Waist- to- stature ratio	Q4 / Q1	2.72	2.03	3.63
	**Elevated diastolic blood pressure¶**			
Body mass index	>85^th^ vs. <85^th^ percentile	1.93	1.36	2.75
Waist- to- stature ratio	Q4 / Q1	1.84	1.43	2.37
	**Elevated body mass index (>85^th^ percentile)**			
Sex	female vs. male	0.81	0.67	0.99
Age group	Child vs. adolescent	0.54	0.45	0.66
Physical activity	(- Others) vs. (+ Others)^7^	1.57	1.24	1.98
	**Subscapular skinfold thickness (>Q3) ‡**			
Sex	female vs. male	2.44	2.04	2.90
Physical activity	(- Others) vs. (+ Others)^7^	2.36	1.98	2.82
	**Suprailiac skinfold thickness (>Q3)**			
Sex	female vs. male	1.99	1.67	2.38
Physical activity	(- Others) vs. (+ Others)^7^	1.97	1.65	2.35
	**Sum of skinfolds – triceps+ subscapular + suprailiac (>Q3)**			
Sex	female vs. male	2.56	2.51	3.05
Physical activity	(- Others) vs. (+ Others)^7^	2.34	1.92	2.85
	**Waist-to-hip ratio (>Q3)**			
Sex	female vs. male	0.38	0.31	0.47
	**Waist-to-height ratio (>Q3)**			
Physical activity	(- Others) vs. (+ Others)††	1.58	1.30	1.93
Age group**	Child vs. adolescent	0.56	0.42	0.75
Sex**	female vs. male	0.81	0.60	1.09

^*^Significant (p<0,001)

** Non significant

** Non significant

† Q1 = lower quartile (or interval) of the distribution of the independent variables; Q4 = upper quartile (or interval) of the distribution of independent variables;

‡ Q3 = upper tertile (or interval) of the distribution of the independent variables.

§ Elevated total cholesterol: > 200mg/dL Elevated LDL-C: > 130mg/dl, Desirable levels of HDL-C: up to 10 years of age ≥ 40 mg/dL; 10 to 18 ≥35 mg/dL^25^

¶ >90^th^ percentile

†† less (physically) active than the other students versus more active than the other students

Students with “excess weight” (BMI > 85th percentile) presented 3.25 (2.38 - 4.45) times higher odds to have high systolic blood pressure and 1.93 (1.36-2.75) times higher odds to have high diastolic blood pressure. Those students enrolled from public schools presented 2.48 (1.75-3.54) times more odds to have an elevated systolic blood pressure than those enrolled from private schools.

Considering LDL-C, the OR of a student with increased “sum of the three skinfold measurements”, to have elevated levels of LDL-C (>130 mg/dL) was 2.34 (1.79-3.06) times higher than the odds of those with normal values for this variable. Those students with a BMI value higher than the 85^th^ percentile had 1.95 (1.46-2.62) times higher odds to have “undesirable levels of HDL-C” than others. The OR of a student with increased WHR, to have “undesirable levels of HDL-C” was 2.56 (1.99-3.29).

Students considered to be “less active than the others” presented 1.58 (1.30 – 1.93) and 1.57 (1.24 – 1.98) times more odds to have respectively increased WHtR values and “excess weight” (BMI > 85^th^ percentile) when compared with students considered to be “more active than the others”. Being female [0.81 (0.60-1.09); 0.81 (0.67-0.99)] and child [0.56 (0.42 – 0.75); 0.54 (0.45 – 0,66)] was “protective” for undesirable conicity shape (increased WHtR values) and “excess weight” (elevated BMI) in comparison of being male and adolescent.

Female students presented higher odds to have increased subscapular, suprailiac and sum of skin fold thickness measurements than male students. Whereas, more male students were found to present more odds to have increased WHR e WHtR values than female students. More adolescents presented higher odds to have increased WHtR values than children students.

Increased WHR was not associated with adiposity and physical activity variables.

Students considered to be “less active than the others” presented 1.58 (1.30-1.93) times more chances to have higher WHtR values than students considered to be “more active than the others“. No significant difference was observed in a multivariate analysis between the groups for other variables.

**Figure 2:**
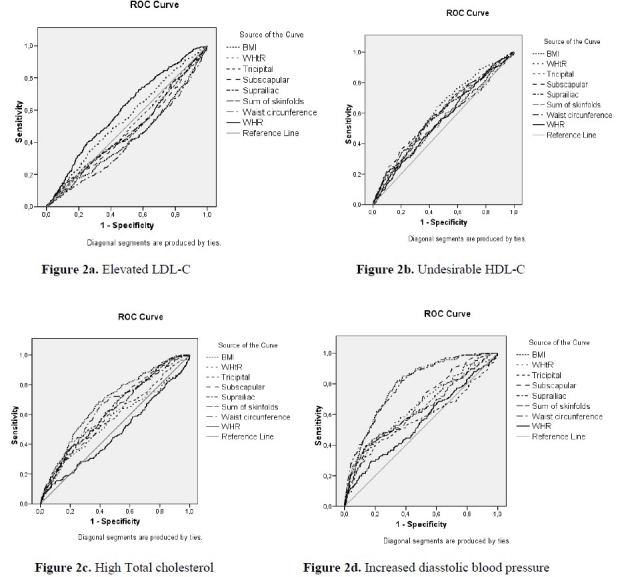
Receiver operating curve analysis and the area under curve of anthropometric parameters in association with cardiovascular risk factors: the Three Cities Heart Study

ROC analyses (Figure 2) showed that the AUC of WHtR was the largest for increased total cholesterol, the AUC of BMI was the largest for increased diastolic blood pressure, while of WC had the largest AUC for increased systolic blood pressure, the AUC of skinfolds sum and WHR were the largest for increased LDL-C and low HDL-C.

The cutoff points for each anthropometric index, with their respective sensibility and specificity were defined as the point of greatest sum value of sensibility and specificity together with less false-positives results (1-Specificity). BMI values above 19.58Kg/m^2^ were indicative of elevated LDL-C with 53.8% sensibility and 58,5% specificity, while WHtR values above 0.435 were indicative of increased LDL-C levels, with 72.2% sensibility and 45.5% specificity.

## DISCUSSION

While some published papers have compared the correlation between CVD risk factors and anthropometric indices, relying on multiple regression to select the best indices for CVD risk factors prediction,^15^,^16^ they bring up the problem of multicollinearity, since the highly correlated indices could invalidate the results.

We found stronger correlation coefficients values between CVD risk factors and anthropometric parameters, compared to Ville Santé II study.^24^ In our study, the strongest correlation coefficients values were documented between systolic and diastolic blood pressure with WC (0.45 and 0.31), and BMI (0.42 and 0.29), all being stronger than those found in Ville Santé II study. However, except for total cholesterol and systolic blood pressure, our correlation coefficients values between WHtR and other CVD risk factors were slightly smaller (0.17 for total cholesterol, -0.04 for HDL-C, 0.15 for LDL-C, 0.11 and 0.15 for diastolic and systolic blood pressure) than that from the Ville Santé II study (total cholesterol: 0.03 in males and 0.14 in females, HDL-C: -0.32 in males and -0.23 in females, LDL-C: 0.16 in males and 0.19 in females, diastolic blood pressure: 0.14 in males and 0.26 in females, systolic blood pressure: 0.16 in males and 0.12 in females).

Studying a sub sample (4 to 17 years of age) from the third National and Nutrition Examination Survey (NHANES III), Khan et al.^28^ found slightly higher WHtR mean values (0.46, SE 0.00) to that of this Brazilian population (0.45, SE 0.00), while another study^29^ found slightly smaller values (0.42, SD 0.04)than ours.

It is demonstrated that, among the main CVD risk factors, adverse serum lipid profile had the highest role for the myocardial infarction global rate.^30^

The results of the present anthropometric indices showed weak to good predictability for the studied CVD risk factors, and although the anthropometric indices with the largest AUC were identified, it should be noted that, as found by others,^31^,^32^ the differences in the AUC for the four anthropometric indices were often small with overlapping 95% confidence intervals.

WHtR had the largest AUC for high total cholesterol levels, the second largest AUC for undesirable HDL-C levels, and the fourth largest AUC for high LDL-C levels, while the sum of the three skin folds presented the largest AUC for high LDL-C levels. WHR was slightly better than WHtR in predicting undesirable HDL-C levels.

BMI presented the largest AUC for increased DBP and followed for increased SBP. The time spent on watching television presented the largest AUC for WC.

The results of this study suggest that for children and adolescents, the following cutoffs: BMI=19, Kg/m^2^, subscapular skinfold =11mm, suprailiac skinfold =15mm WC=73cm, triceps skinfold =12mm, sum of three skinfolds = 40mm, WHR=0.80 and WHtR = 0.44.

Considering the lipid profiles, the present study found an optimal WHtR cutoff value of 0.44 for children and adolescents.

Consistent with some other studies, we found that WHtR could serve better than the other studied anthropometric variables and indices for identifying adverse concentrations of total cholesterol, and a good one for undesirable HDL-C levels.

As reported in other studies we found no significant difference in WHtR values according to age and gender.^28^

In populations with a wide range of heights, WHtR may be a more appropriate measurement of adiposity distribution than any single WC cutoff value. Therefore, WHtR is likely to be more robust than WC for assessment of abdominal fat deposition.

In adults, it has been demonstrated that WHtR is a good indicator of abdominal visceral fat, predictor of cardiovascular risk factors and mortality, both in men and women.^15^,^33^

While both WHtR and BMI require height measurement, if this measurement (height) is inaccurate the error will be squared in computing the BMI. Since WHtR does not require specification of gender and age, we suggest that it might replace or supplement the use or gender and age-specific BMI percentiles for assessment of cardiovascular risk associated with overweight or central obesity.

Since a single WC cutoff value would be not appropriated for different populations and also would understate risk in the very short and overstate it in the very tall, this study support the appropriateness of a simple health promotion message to be given to the public that “one’s waist measurement should not exceed half the stature for children and adolescents”, providing an everyone’s individualized cutoff waist measurement.^34^

## CONCLUSION

In our findings, WHtR was the best predictor of high cholesterol followed by undesirable HDL-C values, while BMI better predicted elevated blood pressure among children than local measurements of adiposity.

The mean WHtR value founded among this Brazilian school children and adolescents sample was in a level (0.44) still according to the health message of “keep your waist circumference to less than half your weight” for avoiding health risks. So, according our results, this simple message could also help our health professional dealing with pediatric age group in excess weight prevention efforts.

In conclusion, WHtR is a good predictor of adverse lipid profile among children and adolescents. Its application in the pediatric age group could offer new approaches for reining in adverse health outcomes of the obesity epidemic such as undesirable serum lipid levels.
